# Traumatic Anterior Dislocation of Hip in a Child - Case Report

**DOI:** 10.5704/MOJ.1503.003

**Published:** 2015-03

**Authors:** S Ahmad, P Devkota, KG Mamman

**Affiliations:** Department of Orthopaedics and Trauma Surgery, Suri Seri Begawan Hospital Kuala Belait, Brunei Darussalam; * Department of Orthopaedics and Trauma Surgery, RIPAS Hospital, Bandar Seri Begawan, Brunei Darussalam

**Keywords:** Hip, anterior dislocation, child, trauma

## Abstract

Traumatic hip dislocation in children is relatively rare accounting for about 5% of all hip dislocations. Most of the hip dislocations seen in children are of the posterior type but the much rarer anterior and anterior-inferior (obturator) types have also been described. We present the case of an eight years old girl with an obturator type of hip dislocation following trivial trauma. She was treated with closed reduction and immobilisation in skin traction for three weeks. She was followed up closely for one year and did not develop any complications during that period.

## Introduction

Traumatic hip dislocation in children is relatively a rare injury accounting for about 5% of all hip dislocations^1^. Most of the hip dislocations seen in children are of the posterior type but the much rarer anterior and anterior-inferior (obturator) types have also been described. We report the case of an eight years old girl who presented to the accident and emergency department with this rare injury following a fall.

## Case Report

An eight years old girl was brought to the Accident and Emergency department following a fall whilst playing with friends. She had a painfully deformed right hip with the right leg fixed in abduction and external rotation, and unable to bear weight

**Fig. 1:**
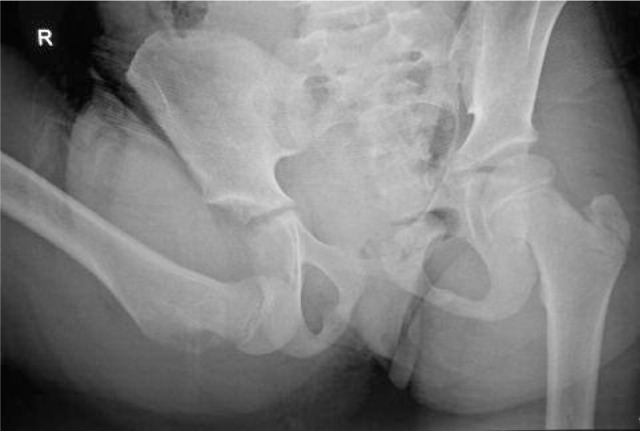
Plain radiograph of the child showing anterior obturator dislocation of right hip.

**Fig. 2:**
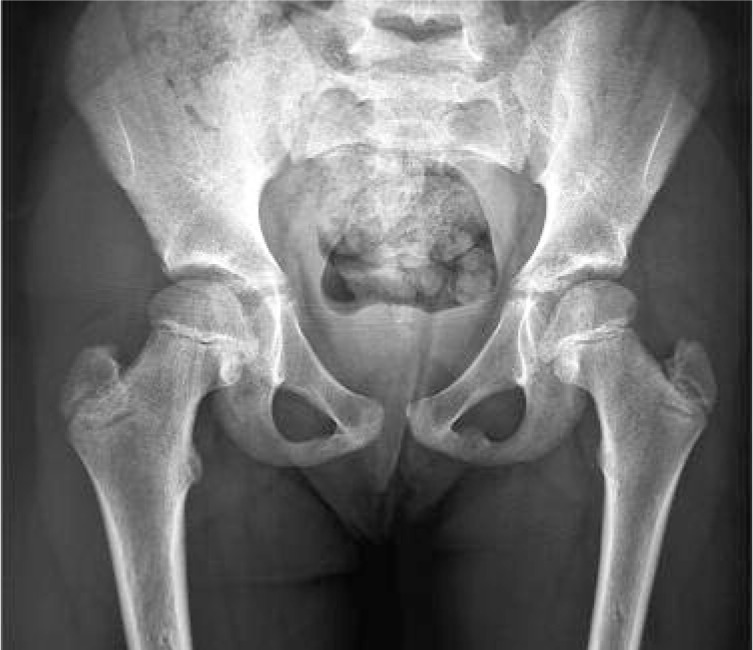
Post reduction radiograph showing good reduction of the hip.

On examination the right hip was fixed in flexion, abduction and external rotation. There was no neurological deficit and the distal pulses were palpable. The radiographs of the pelvis revealed a hip dislocation with the femoral head lying inferior to the acetabulum, in the obturator foramen (Figure 1). Under general anaesthesia, within six hours of injury, the dislocation was reduced by traction, hyperabduction, external rotation and adduction and internal rotation after the femoral head was cleared from the obturator foramen. Postreduction radiographs confirmed the femoral head in the acetabulum without any fractures (Figure 2) The right lower limb was immobilized in skin traction for three weeks

On discharge from the hospital after three weeks, the patient had painless, full range movements of the left hip. She was discharged home with axillary crutches to allow weight bearing as tolerated. Radiographs of the pelvis showed a well reduced right femoral head. The patient was reviewed at three and six months.. She had a painless right hip and was ambulating unassisted full weight bearing. Radiographs showed a well reduced femoral head with no signs of avascular necrosis (AVN).

**Fig. 3:**
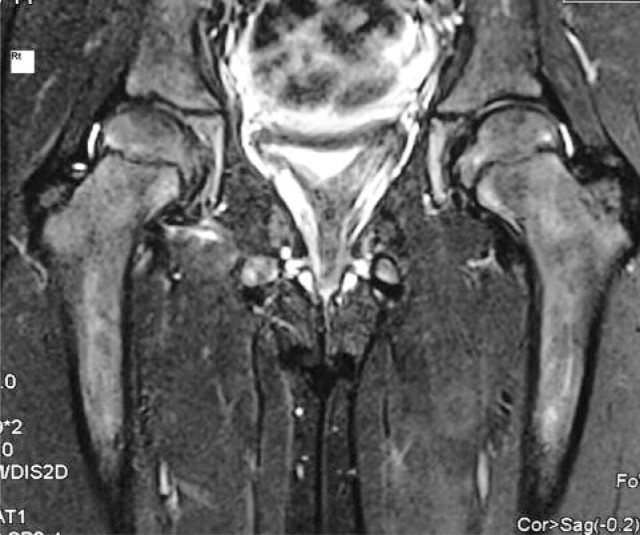
Magnetic resonance image (MRI) of the right hip after one year showed no evidence of avascular necrosis (AVN).

At one year follow-up, the patient had painless full-range movements in the right hip with no signs of AVN on plain radiographs as well as on magnetic resonance image (Figure 3)

## Discussion

Traumatic hip dislocation in children is relatively a rare injury accounting for about 5% of all hip dislocations. Most of the hip dislocations seen in children are of the posterior type but the much rare anterior type has also been described accounting for about 5% to 10% of all paediatric hip dislocations^2,3^ The anterior-inferior (obturator) type has been reported in less than five cases in the English literature1. This dislocation can be caused by high or low energy trauma^2,3^ Complications include associated fractures (40%)^4^, neurological and vascular compromise (25%)^3^, AVN (10%)^3,4,5^ and articular cartilage injury (6%)^2^.

This injury should be treated as an emergency and reduced within six hours of injury, as a delayed reduction predisposes to AVN^3^. Although the outcome of AVN in children is similar to that in adults, the complication is more devastating in children as treatment options for AVN present a challenge in children^3^. To reduce the risk of AVN, reduction should be carried out as early as possible with sufficient sedation and analgesia to lower the risk for physeal injury^3^.

After an early reduction and activity modification our patient recovered well with no signs of AVN or physeal injury after one year and parents were advised for an annual follow-up of the child with MRI scan and modification of lifestyle.
